# Derivation of economic values for production traits in aquaculture species

**DOI:** 10.1186/s12711-016-0278-x

**Published:** 2017-01-05

**Authors:** Kasper Janssen, Paul Berentsen, Mathieu Besson, Hans Komen

**Affiliations:** 1 Animal Breeding and Genomics, Wageningen University and Research, Droevendaalsesteeg 1, 6708 PB Wageningen, The Netherlands; 2 Business Economics Group, Wageningen University and Research, Hollandseweg 1, 6706 KN Wageningen, The Netherlands; 3 Génétique Animale Biologie Intégrative, INRA, AgroParisTech, Université Paris-Saclay, 78350 Jouy-en-Josas, France

## Abstract

**Background:**

In breeding programs for aquaculture species, breeding goal traits are often weighted based on the desired gains but economic gain would be higher if economic values were used instead. The objectives of this study were: (1) to develop a bio-economic model to derive economic values for aquaculture species, (2) to apply the model to determine the economic importance and economic values of traits in a case-study on gilthead seabream, and (3) to validate the model by comparison with a profit equation for a simplified production system.

**Methods:**

A bio-economic model was developed to simulate a grow-out farm for gilthead seabream, and then used to simulate gross margin at the current levels of the traits and after one genetic standard deviation change in each trait with the other traits remaining unchanged. Economic values were derived for the traits included in the breeding goal: thermal growth coefficient (*TGC*), thermal feed intake coefficient (*TFC*), mortality rate (*M*), and standard deviation of harvest weight ($\sigma_{HW}$). For a simplified production system, improvement in *TGC* was assumed to affect harvest weight instead of growing period. Using the bio-economic model and a profit equation, economic values were derived for harvest weight, cumulative feed intake at harvest, and overall survival.

**Results:**

Changes in gross margin showed that the order of economic importance of the traits was: *TGC*, *TFC*, *M*, and $\sigma_{HW}$. Economic values in € (kg production)^−1^ (trait unit)^−1^ were: 0.40 for *TGC*, −0.45 for *TFC*, −7.7 for *M*, and −0.0011 to −0.0010 for $\sigma_{HW}$. For the simplified production system, similar economic values were obtained with the bio-economic model and the profit equation. The advantage of the profit equation is its simplicity, while that of the bio-economic model is that it can be applied to any aquaculture species, because it can include any limiting factor and/or environmental condition that affects production.

**Conclusions:**

We confirmed the validity of the bio-economic model. *TGC* is the most important trait to improve, followed by *TFC* and *M*, and the effect of $\sigma_{HW}$ on gross margin is small.

## Background

In Europe, over 80% of aquaculture production originates from breeding programs, which in most cases apply family selection with the aim of improving multiple traits simultaneously [1]. Breeding goal traits are often weighted based on desired gains rather than economic values [2], which compromises economic gain [3, 4].

In aquaculture species, economic values are available only for a few species, although their importance has repeatedly been underlined, e.g. [5, 6]. Profit equations have been used to derive economic values for Nile tilapia (*Oreochromis niloticus*) [7], common carp (*Cyprinus carpio*) [8], Australian abalones (*Haliotis rubra* and *H. laevigata*) [9], and crayfish (*Cherax tenuimanus*) [10]. Besson et al. [11, 12] used a bio-economic model to derive economic values for African catfish (*Clarias gariepinus*) that were produced in a land-based aquaculture system in which water is treated and recirculated and for growth rate in European seabass (*Dicentrarchus labrax*) under varying temperature conditions, respectively.

For livestock species with simple and highly controlled production systems, such as pig production, economic values can be derived from a profit equation, e.g. [13]. For production systems with a higher degree of complexity, partly due to seasonal variation, such as in dairy cattle and sheep farming, profit equations may fail to provide an adequate description of the farming system and bio-economic models are required [14–16]. In general, bio-economic models provide a more accurate description of farming systems than profit equations and are, therefore, increasingly used to estimate economic values [2].

Fish farms are complex production systems for two reasons. First, fish are kept outdoors in most farming systems and, thus, are exposed to fluctuating environmental conditions. Seasonal variation in temperature causes variation in growth rate of fish, because of their ectothermic nature. Fish are harvested at a constant weight rather than at a constant age, hence the length of a production cycle depends on the stocking date. Second, production output of a farm is determined by constraints such as oxygen availability [11] and stocking density. Stocking density constrains production output for many important aquaculture species, including Atlantic salmon (*Salmo salar*), European seabass, and gilthead seabream (*Sparus aurata*). Thus, bio-economic models could prove useful to derive economic values for aquaculture species.

The objectives of this study were: (1) to develop such a bio-economic model, (2) to apply the model to determine the economic importance and economic values for various traits in a case-study on gilthead seabream, and (3) to validate the model by comparison with a profit equation for a simplified production system.

## Methods

### Traits

The breeding goal considered here includes growth rate, feed intake rate, mortality rate, and uniformity in harvest weight. Growth rate affects revenues, feed costs and juvenile costs; feed intake rate affects feed costs; mortality rate affects feed costs and juvenile costs. Feed and juveniles are major costs in production [17], thus including growth and feed intake in the breeding goal is common practice in livestock [18, 19]. Uniformity, i.e. size variation around the mean harvest weight, determines the distribution of fish over price categories at harvest, and thus affects revenues via the average sales price of fish.

Economic values are specific for the unit in which a trait is expressed [20]. Here, growth rate is expressed in units of thermal growth coefficient (*TGC*) [21]. *TGC* is a standardized measure of growth in fish that takes stocking weight and temperature variation over the lifespan of a fish into account. *TGC* is widely used [22], is more accurate than other measures of growth rate [23], and is relatively robust to differences in temperature regimes [24, 25]. Feed intake is assumed to be determined by the same variables as bodyweight and gain in bodyweight, because energy requirement is largely determined by bodyweight and gain in bodyweight [26]. In this study, feed intake rate is, therefore, expressed in units of thermal feed intake coefficient *TFC*, a *TGC* analogue. *TFC* takes stocking weight and temperature variation over the lifespan of a fish into account. The *TFC* model is independent of the *TGC* model, i.e. a modelled change in growth rate will not affect modelled feed intake rate and vice versa, which is a prerequisite to derive economic values. Mortality rate (*M*) is expressed as % of mortality per day. Uniformity is expressed as the standard deviation of harvest weight ($\sigma_{HW}$) in grams.

### Bio-economic model

**Fig. 1:**
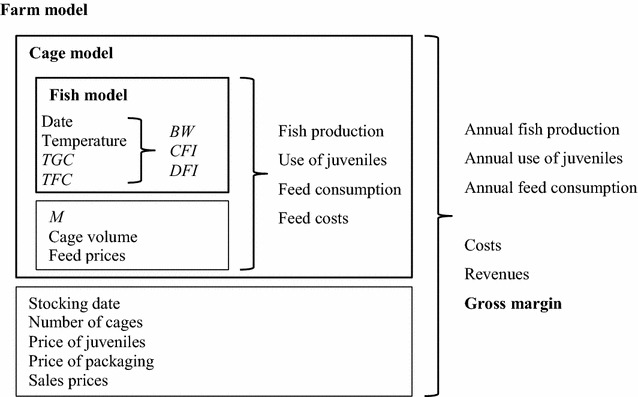
Schematic overview of the bio-economic model. *TGC* thermal growth coefficient, *TFC* thermal feed intake coefficient, *BW* bodyweight, *CFI* cumulative feed intake, *DFI* daily feed intake, *M* mortality rate

The bio-economic model developed by Besson et al. [12] was adapted to simulate production systems with seasonal variation in temperature and in which density constrains production output, in this case a typical grow-out farm for gilthead seabream in Greece. The model is a deterministic simulation model that is programmed in R version 2.12.2. [27]. The model simulates operation of the farm during an average year. The farm consists of 20 cages of 2800 m^3^ each and produces about 550 tons annually. As shown in Fig. 1, the model consists of three hierarchical parts: a fish model, a cage model, and a farm model. Inputs into the fish model are: stocking date, temperature coefficients, *TGC*, and *TFC*. Outputs of the fish model for each stocking date are: bodyweight per day per fish, feed consumption per day per fish, and harvest date. Inputs into the cage model are: outputs of the fish model, *M*, cage volume, and feed prices. Outputs of the cage model for each stocking date are per production cycle of a cage: fish production, number of juveniles stocked, feed consumption, and feed costs. A production cycle is the period between stocking and harvesting a cage. Inputs of the farm model are: outputs of the cage model, number of cages, price of juveniles, price of packing, and sales prices. Outputs of the farm model are total per year: fish production, number of juveniles stocked, feed consumption, feed costs, juvenile costs, packing costs, revenues from fish sales, and gross margin.

The model was used to derive economic values of the traits mentioned above. Economic values give the expected change in profit from a small change in trait level, keeping the level of all other traits constant. Genetic change does not affect fixed costs, hence change in profit due to genetic change equals change in gross margin. When change in trait level equals the additive genetic standard deviation ($\sigma_{A}$) [15], the resulting change in gross margin indicates how important that trait is, because $\sigma_{A}$ is indicative of the rate at which breeding values can be improved [28]. To determine the relative importance of the traits and to derive economic values, the model was run under two situations: first before genetic change and second, after a change of one $\sigma_{A}$ in one trait with the other traits kept constant. For the trait ‘uniformity’, minimum and maximum values of the possible range of $\sigma_{A}$ were used, because the actual value was unknown. Trait levels were changed in the desired direction of the genetic change. Economic values were expressed per kg of fish produced in the situation before genetic change [29] and were calculated as:

1 $$Economic\;value = {{\left( {\frac{{gross\;margin_{A} - gross\;margin_{B} }}{{trait\;level_{A} - trait\;level_{B} }}} \right)} \mathord{\left/ {\vphantom {{\left( {\frac{{gross\;margin_{A} - gross\;margin_{B} }}{{trait\;level_{A} - trait\;level_{B} }}} \right)} {fish \;production_{B} }}} \right. \kern-0pt} {fish \;production_{B} }},$$

where subscripts indicate before (*B*) and after (*A*) genetic change.

### Model equations

The “Estimation of model coefficients” section describes the derivation of model coefficients from farm data. These coefficients (see Table 1) are used as the equations’ coefficients of the bio-economic model. The three parts of the bio-economic model are described in the following three subsections.

**Table 1:** Estimated coefficients used in model equations

Symbol	Meaning	Value	Standard error	Unit
$TM$	Annual mean temperature	19.57	0.0119	°C
$TA$	Amplitude of temperature	−4.806	0.0167	°C
$t_{A}$	Date at which temperature equals TM	−32.49	0.2057	Day
$TGC$	Thermal growth coefficient	12.6	0.0847	g^2/3^/(day degrees · 1000)
$TFC$	Thermal feed intake coefficient	8.25	0.157	g^0.544^/(day degrees · 1000)
$p$	Weight exponent to predict cumulative feed intake	0.544	0.00282	–
$M$	Mortality rate	0.0300	0.000164	%/day

### Estimation of model coefficients

Coefficients in the equations to describe temperature, fish growth, feed intake, and number of fish per cage were derived from recent farm data of the company Andromeda S.A., which is hereafter referred to as ‘data’. The data included daily records of temperature, feed provided, and mortality, and regular records of bodyweight from 15 cages of a farm located in Vonitsa, Northern Greece during the period 2013 through 2015.

Seasonal variation in daily water temperature throughout the year followed a sinusoidal pattern. Therefore, the equation to describe daily temperature ($T_{{t_{s,a} }}$) in °C was [30]:

2 $$T_{{t_{s,a} }} = TM - TA \cdot sin\left( {2\pi \cdot \left( {t_{s,a} - t_{A} } \right)/365} \right),$$

where *TM* is the average annual temperature (°C), *TA* is the range of temperatures around *TM* (°C), $t_{A}$ is the time of the year at which $T_{{t_{s,a} }}$ equaled *TM*, and $t_{s,a}$ represents the date defined as:

3 $$t_{s,a} = s + a,$$

where stocking date *s* ($s = 1, \ldots , n$) equals 1 on January 1 2013 and *a* ($a = 0, \ldots , n$) is the age of the fish (days). To estimate $TM$, $TA$, and $t_{A}$, Eq. 2 was fitted to the data by means of non-linear least-squares regression in R. Table 1 shows the resulting coefficients.

Bodyweight in seabream can be predicted from the stocking weight and the sum of daily effective temperatures. Daily effective temperature is the daily temperature minus 12 °C, where 12 °C represents the minimum temperature for seabream growth [31]. Therefore, the equation to describe bodyweight (in g) at $t_{s,a} \;(BW_{{t_{s,a} }} )$ was [32]:

4 $$BW_{{t_{s,a} }} = \left( {BW_{{t_{s,0} }}^{2/3} + \frac{TGC}{1000} \cdot \mathop \sum \limits_{{i = t_{s,0} }}^{{(t_{s,a} ) - 1}} \left( {T_{i} - 12} \right) } \right)^{3/2} ,$$

where $BW_{{t_{s,0} }}$ is bodyweight at stocking (in g), and $\sum\nolimits_{{i = t_{s,0} }}^{{(t_{s,a} ) - 1}} {\left( {T_{i} - 12} \right)}$ represents the sum of effective temperatures (day degrees) over the lifespan of a fish excluding $t_{s,a}$. To estimate $TGC$, Eq. 4 was fitted to the data by means of non-linear least-squares regression in R. Instead of fixing exponents 2/3 and 3/2, fitting these to the data resulted in values of 0.612 and 1/0.612 but this barely improved accuracy of the model. Values of 2/3 and 3/2 were preferred, because standardization of growth models allows for a better comparison of growth rate across studies. Analogous to $BW_{{t_{s,a} }}$, the model to describe cumulative feed intake (in g) at $t_{s,a}$ ($CFI_{{t_{s,a} }}$) was:

5 $$CFI_{{t_{s,a} }} = \left( {BW_{{t_{s,0} }}^{p} + \frac{TFC}{1000} \cdot \mathop \sum \limits_{{i = t_{s,0} }}^{{t_{s,a} }} \left( {T_{i} - 12} \right)} \right)^{1/p} - BW_{{t_{s,0} }} ,$$

where $p$ is a weight exponent, and $\sum\nolimits_{{i = t_{s,0} }}^{{t_{s,a} }} {\left( {T_{i} - 12} \right)}$ represents the sum of effective temperatures (day degrees) over the lifespan of a fish, including $t_{s,a}$. The term $BW_{{t_{s,0} }}$ was subtracted from $\left( {BW_{{t_{s,0} }}^{p} + \frac{TFC}{1000} \cdot \sum\nolimits_{{i = t_{s,0} }}^{{t_{s,a} }} {\left( {T_{i} - 12} \right)} } \right)^{1/p}$ to force the model through the intercept. To estimate $TFC$ and $p$, Eq. 5 was fitted to the data by means of non-linear least-squares regression in R. Parameter $M$ (%/day) was assumed to be constant over time, hence the number of fish alive decreased exponentially in time. The model to describe the number of fish at $t_{s,a}$ ($N_{{t_{s,a} }}$) was:

6 $$N_{{t_{s,a} }} = N_{{t_{s,0} }} \cdot \left( {1 - \frac{M}{{100{\text{\% }}}}} \right)^{{\left( {t_{s,a} } \right) - 1}} ,$$

where $N_{{t_{s,0} }}$ is the number of fish stocked. To estimate $M$, Eq. 6 was fitted to the data by means of non-linear least-squares regression in R.

### Fish model

Date ($t_{s,a}$) was modelled as in Eq. 3. Temperature ($T_{{t_{s,a} }}$) was modelled as in Eq. 2. Bodyweight ($BW_{{t_{s,a} }}$) was modelled as in Eq. 4, where stocking weight ($BW_{{t_{s,0} }}$) was 4.4 g, equal to the average in the data. Equation 4 was rewritten to calculate the harvest date $(t_{s,h}$) as:

7 $$\mathop \sum \limits_{{i = t_{s,0} }}^{{(t_{s,h} ) - 1}} \left( {T_{i} - 12} \right) = \frac{{BW_{{t_{s,h} }}^{2/3} - BW_{{t_{s,0} }}^{2/3} }}{TGC/1000},$$

where $BW_{{t_{s,h} }}$ is the average harvest weight, here set to the desired market weight of 400 g. Solving the right hand side of the equation, yields $\sum\nolimits_{{i = t_{s,0} }}^{{\left( {t_{s,h} } \right) - 1}} {\left( {T_{i} - 12} \right)} = 4084$ day degrees. Cumulative feed intake $\left( {CFI_{{t_{s,a} }} } \right)$ was modelled as in Eq. 5. $CFI_{{t_{s,h} }}$ was set equal to $CFI_{{(t_{s,h} ) - 1}}$, because fish are not fed on the day that they are harvested. Daily feed intake ($DFI_{{t_{s,a} }}$) was modeled as:

8 $$DFI_{{t_{s,a} }} = CFI_{{\left( {t_{s,a} } \right) + 1}} - CFI_{{t_{s,a} }} .$$

### Cage model

To maximize production, standing stock in a cage reaches the maximum allowable density of 15 kg/m^3^ at harvest. For the 2800 m^3^ cages, per production cycle fish production is thus 42,000 kg or 100,500 fish. To compensate for mortality, a larger number of fish is stocked than harvested. The number of fish in a cage at $t_{s,a}$ ($N_{{t_{s,a} }}$) was modeled as:

9 $$ N_{{t_{s,a} }} = 100,500 \cdot \left( {1 - \frac{M}{{100{\text{\% }}}}} \right)^{{\left( {t_{s,h} - t_{s,a} } \right)}} . $$

The number of juveniles stocked per cage ($$ N_{{t_{s,0} }} $$) was calculated by substituting $$t_{s,a}$$ for $$t_{s,0}$$. Daily feed intake per cage (kg) at $$t_{s,a}$$ ($$DFIcage_{{t_{s,a} }} )$$ was modeled as:

10 $$DFIcage_{{t_{s,a} }} = N_{{t_{s,a} }} \cdot DFI_{{t_{s,a} }} /1000.$$

Total feed consumption per cage (kg) stocked at date $t_{s,0}$ ($TFIcage_{{t_{s,0} }}$) was calculated as:

11 $$TFIcage_{{t_{s,0} }} = \mathop \sum \limits_{{i = t_{s,0} }}^{{t_{s,h} }} DFIcage_{i} .$$

Depending on bodyweight, fish are fed different feed types. Daily feed costs per cage $(DFCcage_{{t_{s,a} }} )$ were calculated as the product of $DFIcage_{{t_{s,a} }}$ and feed price per size category. Table 2 shows the price of feed types for the fish size categories. Total feed costs per cage (€) stocked at date $t_{s,0}$ ($TFCcage_{{t_{s, 0} }}$) were calculated as:

12 $$TFCcage_{{t_{s, 0} }} = \mathop \sum \limits_{{i = t_{s,0} }}^{{t_{s,h} }} DFCcage_{i} .$$

**Table 2:** Feed price per fish size category in 2014 [Personal communications Andromeda S.A., 2015]

Fish size (g)	Price (€/kg)
<7	2.21
7–13	1.97
13–30	1.65
30–80	1.26
80–300	1.12
>300	1.17

### Farm model

For the farm model, the cage model was run repeatedly over the whole range of stocking dates, from 1 (January 1th) to 365 days (December 31th) by one-day steps. Per stocking date, age at harvest $\left( {t_{s,h} - t_{s,0} } \right)$, $TFIcage_{{t_{s,0} }}$, $TFCcage_{{t_{s, 0} }}$, and $N_{{t_{s,0} }}$ were calculated. These results were averaged over all stocking dates to compute the average production cycle of a cage. The period between two successive production cycles is three days [Personal communications Andromeda S.A., 2015]. For the 20 cages that are present on the farm, the number of production cycles per year was calculated as:

13 $$Production\;cycles\;per\;year = 20 \cdot \frac{365}{{average\left( {t_{s,h} - t_{s,0} } \right) + 3}}.$$

Average results per production cycle were multiplied by the number of production cycles per year to compute outputs at the farm level per year: fish production, number of juveniles stocked, feed consumption, and feed costs. Juvenile costs at the farm level were calculated by multiplying the number of juveniles stocked by the price of juveniles of €0.20 per piece. Packing costs at the farm level were calculated by multiplying fish production by the price of packing of €0.33 per kg fish. Revenues from fish sales at the farm level were calculated as the product of fish production and average sales price. Average sales price was computed as the proportion of fish of each category in Table 3 multiplied by its corresponding sales price. Three percent of the fish harvested are deformed. The size distribution of the remaining fish was calculated from a normal probability density function with $\mu = 400$ and $\sigma_{HW} = 60$ [Personal communications Andromeda S.A., 2015].

**Table 3:** Sales price per fish size category in 2014 [Personal communications Andromeda S.A., 2015]

Category (g)	Price (€/kg)
<100	0
100–200	1.65
200–300	4.15
300–400	4.52
400–600	4.63
>600 g	5.27
Deformed	2.52

### Additive genetic standard deviation of traits

The economic importance of each trait in the breeding goal depends on the change in gross margin, which itself depends on the change in trait level of one $\sigma_{A}$. The genetic coefficient of variation ($CVA$) can be used to estimate $\sigma_{A}$ from the mean trait level ($\mu$) [28]:

14 $$\sigma_{A} = \frac{{CV_{A} }}{{100{\text{\% }}}} \cdot \mu .$$

For $TFC$, $\sigma_{A}$ can be estimated from the genetic variation in $BW_{{t_{s,h} }}$. For this study, $BW_{{t_{s,h} }}$ was set equal to 400 g, and $\sum\nolimits_{{i = t_{s,0} }}^{{(t_{s,h} ) - 1}} {\left( {T_{i} - 12} \right)}$ was 4084 day degrees (Eq. 7). $CVA$ of bodyweight was estimated to be 10.6% based on data from Navarro et al. [33]. For $BW_{{t_{s,h} }}$, $\sigma_{A}$ was thus 42.4 g. The distribution of $BW_{{t_{s,h} }}$ was simulated in R as $BW_{{t_{s,h} ,n}} = \mu + z_{n} \cdot \sigma_{A}$, where $\mu = 400$ and $\sigma_{A} = 42.4$, and $z_{n}$ is a standard normal distribution ($z_{n} \sim N\left( {0,1} \right)$) with $n = 1, \ldots , 10^{6}$. From this simulation, $\sigma_{A}$ of $TGC$ was estimated as (Appendix 1):

15 $$\begin{aligned} \sigma_{A} {\text{of }}TGC & \approx \sqrt {Var(TGC_{n} )} \approx \sqrt {\left( {\frac{1000}{4084}} \right)^{2} \cdot Var\left( {BW_{{t_{s,h} ,n}}^{2/3} } \right)} \\ & = \frac{1000}{4084}\sqrt {\frac{1}{{10^{6} - 1}} \cdot \mathop \sum \limits_{i = 1}^{n} \left( {BW_{i}^{2/3} - 400^{2/3} } \right)^{2} } \\ & = 0.95 {\text{ g}}^{2/3} /\left( {{\text{day}}\;{\text{degrees }} \cdot 1000} \right). \\ \end{aligned}$$

For $TFC$, $\sigma_{A}$ can be estimated from the genetic variation in both $BW_{{t_{s,0} }}$ and $CFI_{{t_{s,h} }}$. For $CFI_{{t_{s,h} }}$, $\sigma_{A}$ can be approximated by (Appendix 2):

16 $$\sigma_{A} \;{\text{of}}\;CFI_{{t_{s,h} }} \approx \frac{1.75 }{{r_{A} }} \cdot \sigma_{A} \;of\;BW_{{t_{s,h} }} ,$$

where $r_{A}$ is the genetic correlation between $BW_{{t_{s,h} }}$ and $CFI_{{t_{s,h} }}$, which was assumed to be 0.90 [34]. Solving Eq. (16), $\sigma_{A}$ of $CFI_{{t_{s,h} }}$ was equal to 82 g. Based on an average $CFI_{{t_{s,h} }}$ of 713 g in our study, the $CVA$ of $CFI_{{t_{s,h} }}$ was 12%, which is close to values reported for other species [34, 35]. Genetic variances for $CFI_{{t_{s,h} }}$ and $BW_{{t_{s,0} }}$ were simulated to calculate $\sigma_{A}$ of $TFC$. In our study, the average $BW_{{t_{s,0} }}$ was 4.4 g. Based on a $CVA$ of 10.6% for bodyweight, $\sigma_{A}$ of $BW_{{t_{s,0} }}$ was equal to 0.45 g. The distribution of $CFI_{{t_{s,h} }}$ was simulated in R as $CFI_{{t_{s,h} , n}} = \mu + z_{n} \cdot \sigma_{A}$, where $\mu = 713$, $\sigma_{A} = 82$, and $z_{n}$ is a standard normal distribution ($z_{n} \sim N\left( {0,1} \right)$) with $n = 1, \ldots , 10^{6}$. The distribution of $BW_{{t_{s,0} }}$ was simulated in R as $BW_{{t_{s,0} ,n}} = \mu + z_{n} \cdot \sigma_{A}$, where $\mu = 4.4$, $\sigma_{A} = 0.45$ and $z_{n}$ is a standard normal distribution ($z_{n} \sim N\left( {0,1} \right)$) with $n = 1, \ldots , 10^{6}$. A covariance of zero was assumed between $CFI_{{t_{s,h} }}$ and $BW_{{t_{s,0} }}$. Based on the simulations, $\sigma_{A}$ of $TFC$ was estimated as:

17 $$\begin{aligned} \sigma_{A} \;{\text{of}}\;TFC & \approx \sqrt {Var(TFC_{n} )} \\ & = \sqrt {Var\left( {1000 \cdot \frac{{\left( {CFI_{{t_{s,h} , n}} + BW_{{t_{s,0} ,n}} } \right)^{p} - BW_{{t_{s,0} ,n}}^{p} }}{4084}} \right)} \\ & = \frac{1000}{4084} \cdot \sqrt {\frac{1}{{10^{6} - 1}} \cdot \mathop \sum \limits_{i = 1}^{n} \left( {\begin{array}{*{20}c} {\left( {CFI_{{i}} + BW_{i} } \right)^{0.544} - BW_{i}^{0.544} } \\ { - \left( {\left( {713 + 4.4} \right)^{0.544} - 4.4^{0.544} } \right)} \\ \end{array} } \right)^{2} } \\ & = 0.55\;{\text{g}}^{0.544} /\left( {{\text{degree}}\;{\text{days }} \cdot 1000} \right). \\ \end{aligned}$$

For $M$, $\sigma_{A}$ can be estimated from the genetic variation in cumulative mortality at harvest ($CM_{{t_{s,h} }}$). The average $CM_{{t_{s,h} }}$ was 14.9%. In animal breeding, an underlying liability scale is commonly used to analyze mortality and survival [36]. Heritability of $CM_{{t_{s,h} }}$ on the liability scale was assumed to be 0.17 [37] and by definition $\sigma_{P}$ is equal to 1, hence $\sigma_{A} = \sqrt {h^{2} } = \sqrt {0.17}$. Before genetic change, the deviation of the threshold from the mean ($x_{B}$) was calculated from the quantile function of a normal distribution in R as $x_{B} = - qnorm\left( {0.149} \right) = 1.04$. After genetic change by one $\sigma_{A}$, the deviation from the threshold from the mean ($x_{A}$) becomes: $x_{A} = x_{B} + \sigma_{A} = 1.04 + \sqrt {0.17} = 1.45$. After genetic change, $CM_{{t_{s,h} }}$ was calculated from the distribution function of a normal distribution in R as:

18 $$\begin{aligned} CM_{{t_{s,h} }} & = \left( {1 - pnorm\left( {x_{A} } \right)} \right) \cdot 100 {\text{\% }} \\ & = \left( {1 - pnorm\left( {1.45} \right)} \right) \cdot 100 {\text{\% }} = 7.34\% . \\ \end{aligned}$$

Average age at harvest was equal to 539 days (Table 4). $M$ after genetic change was calculated as:

$$M = \left( {1 - \left(1- {\frac{{CM_{{t_{s,h} }} }}{100}} \right)^{{\frac{1}{539}}} } \right) \cdot 100$$

19 $$\left( {1 - \left(1- {\frac{7.34}{100}} \right)^{{\frac{1}{539}}} } \right) \cdot 100 = 0.014\% /{\text{day}} .$$

The difference in $M$ before and after genetic change was 0.016, which was treated as the $\sigma_{A}$ of $M$.

Genetic improvement of uniformity reduces the environmental variance of bodyweight. For environmental variance of bodyweight, $CV_{A}$ was calculated as [38]:

20 $$CV_{A} = \frac{{SD\left( {\sigma_{E}^{2} } \right)}}{{\overline{{\sigma_{E}^{2} }} }} \cdot 100{\text{\% ,}}$$

where $SD\left( {\sigma_{E}^{2} } \right)$ is the genetic standard deviation of environmental variance and $\overline{{\sigma_{E}^{2} }}$ is the mean environmental variance. Environmental variance equals phenotypic variance minus genetic variance [39]. The $CV_{A}$ of environmental variance of bodyweight is about 20% in rainbow trout [40] and 41.7% in Atlantic salmon (*Salmo salar*) [41]. For seabream, the actual value was unknown, hence a minimum of 20% and maximum of 40% were used to represent both extremes of the possible range of $\sigma_{A}$. In this study, the trait uniformity was expressed on the standard deviation scale instead of the variance scale. On the standard deviation scale, the $CV_{A}$ is half as large as on the variance scale [42, 43]. For $BW_{{t_{s,h} }}$, $\overline{{\sigma_{E} }} = \sqrt {\sigma_{HW}^{2} - \sigma_{A}^{2} } = \sqrt {60^{2} - 42.4^{2} } = 42.45\;{\text{g}}$. For the minimum $CV_{A}$ of the environmental standard deviation of bodyweight of 10%, $\sigma_{A}$ equals 4.2 g, and for the maximum $CV_{A}$ of the environmental standard deviation of bodyweight of 20%, $\sigma_{A}$ equals 8.5 g.

### Validation of the bio-economic model

To validate the bio-economic model, a simplified production system was assumed for which a profit equation can be developed. In this simplified production system, fish were harvested at a constant sum of effective temperatures instead of constant bodyweight. The sum of effective temperatures at harvest was assumed to be unaffected by genetic change. This allowed a profit equation to be set up as a function of the traits: harvest weight ($BW_{{t_{s,h} }}$), cumulative feed intake at harvest ($CFI_{{t_{s,h} }}$), and survival at harvest ($S_{{t_{s,h} }}$). In the bio-economic model, $S_{{t_{s,h} }} = \frac{{N_{{t_{s,h} }} }}{{N_{{t_{s,0} }} }} \cdot 100{\text{\% }}$. The bio-economic model was adapted by changing the harvest criterion from a bodyweight of 400 g to a sum of effective temperatures of 4084 day degrees. Thus, an increase in $TGC$ resulted in a greater harvest weight instead of a shorter growing period. One $\sigma_{A}$ change in $TGC$ led to change in $BW_{{t_{s,h} }}$; one $\sigma_{A}$ change in $TFC$ led to change in $CFI_{{t_{s,h} }}$; one $\sigma_{A}$ change in $M$ led to change in $S_{{t_{s,h} }}$. Economic values were derived from the bio-economic model using Eq. 1 and trait levels of $BW_{{t_{s,h} }}$, $CFI_{{t_{s,h} }}$, and $S_{{t_{s,h} }}$.

In the profit equation, profit at the farm level was described as:

21 $$Profit = \frac{1000 \cdot Q}{{BW_{{t_{s,h} }} }} \cdot \left( {BW_{{t_{s,h} }} \cdot \frac{sales \;price - packing\;costs}{1000} - CFI_{{t_{s,h} }} \cdot \frac{1}{{0.5 + S_{{t_{s,h} }} /200}} \cdot \left( {\frac{feed \;price}{1000}} \right) - \frac{juvenile \;price}{{S_{{t_{s,h} }} /100}}} \right) - fixed\;costs,$$

where *Q* represents production output of the farm (kg) and was calculated as the product of maximum stocking density, cage volume and number of production cycles per year. $Q$ is not affected by genetic change when the harvest criterion is a sum of effective temperatures of 4084 day degrees. Economic values were calculated as partial derivatives of the profit equation and divided by $Q$ to express them per kg fish production. For $BW_{{t_{s,h} }}$, the economic value was calculated as:

22 $$\begin{aligned} Economic \;value_{{BW_{{t_{s,h} }} }} & = \frac{\delta Profit}{{\delta BW_{{t_{s,h} }} }} \cdot \frac{1}{Q} \\ & = \frac{1000}{{BW_{{t_{s,h} }}^{2} }} \cdot \left( {\frac{{CFI_{{t_{s,h} }} \cdot \left( {feed\;price/1000} \right)}}{{0.5 + S_{{t_{s,h} }} /200}} + \frac{juvenile \;price}{{S_{{t_{s,h} }} /100}}} \right). \\ \end{aligned}$$

For $CFI_{{t_{s,h} }}$, the economic value was calculated as:

23 $$Economic \;value_{{CFI_{{t_{s,h} }} }} = \frac{\delta Profit}{{\delta CFI_{{t_{s,h} }} }} \cdot \frac{1}{Q} = - \frac{feed \;price}{{BW_{{t_{s,h} }} \cdot \left( {0.5 + S_{{t_{s,h} }} /200} \right)}}.$$

For $S_{{t_{s,h} }}$, the economic value was calculated as:

24 $$\begin{aligned} Economic \;value_{S} & = \frac{\delta Profit}{\delta S} \cdot \frac{1}{Q} \\ & = \frac{{CFI_{{t_{s,h} }} \cdot feed \;price}}{{BW_{{t_{s,h} }} \cdot \left( {50 + S_{{t_{s,h} }} + S_{{t_{s,h} }}^{2} /200} \right)}} + \frac{100,000 \cdot juvenile \;price}{{BW_{{t_{s,h} }} \cdot S_{{t_{s,h} }}^{2} }}. \\ \end{aligned}$$

## Results

### Production results before genetic change

Tables 4 and 5 show the results of the model for key production variables and costs, respectively. The annual fish production was about 565 tons and gross margin about 759,000€. Average feed costs were €1.18/kg feed and average sales price was €4.49/kg fish.

**Table 4:** Key production variables of the gilthead seabream farm before genetic change

Item *FCR*	Value
Number of juveniles stocked (year^−1^)	1,659,945
Feed consumption (kg/year)	1,070,177
Fish production (kg/year)	564,661
Cages stocked (year^−1^)	13.4
Average age at harvest (day)	539
Survival (%)	85.1
Biological *FCR* ^a^ (kg feed/kg fish)	1.80
Economic *FCR* ^b^ (kg feed/kg fish)	1.92

^a^Biological *FCR* = feed consumption/(fish production + biomass mortality − biomass juveniles)

^b^Economic *FCR* = feed consumption/fish production

**Table 5:** Economic results for the gilthead seabream farm before genetic change

Item	Farm level (€)	Fish level (€/kg)
Feed costs	1,259,917	2.23
Juvenile costs	331,989	0.59
Packing costs	186,338	0.33
Total variable costs	1,778,244	3.15
Total revenues	2,537,166	4.49
Gross margin	758,922	1.34

### Production results after genetic change and economic values

The effect of the genetic change on production results is illustrated in Table 6. Changes in gross margin show that the order of economic importance of traits was: $TGC$, $TFC$, $M$, and $\sigma_{HW}$. The effect on gross margin of one $\sigma_{A}$ change for each trait relative to the effect of a 8.5 g decrease in *σ*_*HW*_ was 43-fold for *TGC*, 28-fold for $TFC$, and 12-fold for $M$. Non-linearity was strongest for $\sigma_{HW}$ (results not presented for *TGC*, $TFC$, and* M*), for which a doubling of change in trait level from −4.2 to −8.5 g led to 7.7% overestimation of the increase in gross margin.

**Table 6:** Effect of genetic change on production results relative to the situation without genetic change

Trait	Genetic change (trait unit)	Δ Juveniles stocked (year^−1^)	Δ Feed consumption (kg/year)	Δ Fish production (kg/year)	Δ Gross margin (€)
$TGC$ ^a^	+0.95	89,400	−68,409	36,309	213,131
$TFC$ ^b^	−0.55	0	−120,300	0	140,891
$M$ ^c^	−0.016	−137,294	−34,446	0	69,531
$\sigma_{HW}$ ^d^	−4.2	0	0	0	2636
$\sigma_{HW}$	−8.5	0	0	0	4952

^a^Thermal growth coefficient [${\text{g}}^{2/3}$/(day degrees · 1000)]

^b^Thermal feed intake coefficient [${\text{g}}^{0.544}$ /(day degrees · 1000)]

^c^Mortality rate (%/day)

^d^Standard deviation of harvest weight (g)

**Fig. 2:**
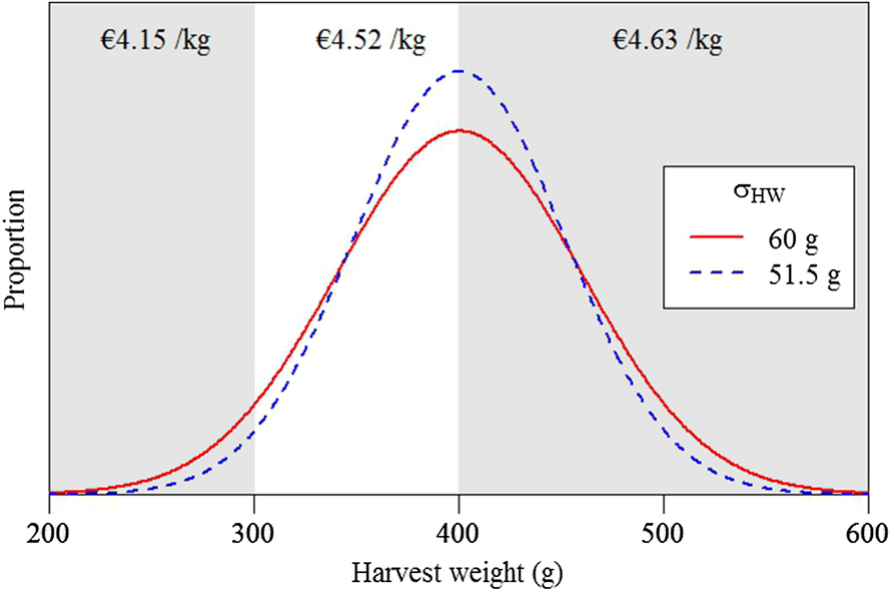
Distribution of harvest weight over sales price categories at different standard deviations of harvest weight (*σ*_*HW*_)

The mechanisms by which changes in trait levels determined changes in gross margin were as follows. An increase in $TGC$ resulted in a lower age at harvest (Eq. 7) and consequently, the number of production cycles per year increased (Eq. 13) and at the farm level, the annual number of juveniles stocked and annual fish production increased. An increase in $TGC$ did not affect daily feed consumption (Eq. 4) and consequently, cumulative feed intake at harvest decreased because the sum of effective temperatures at harvest decreased (Eq. 5) and at the farm level, the annual feed consumption decreased. A decrease in $TFC$ decreased total feed consumption per production cycle (Eqs. 5, 11) but the number of juveniles stocked per production cycle and the fish production per production cycle remained unaltered, thus at the farm level, only annual feed consumption decreased. A decrease in $M$ reduced the number of juveniles stocked per production cycle (Eq. 9) and consequently, daily feed intake per cage decreased because the average number of fish per cage per day was smaller (Eq. 10), but fish production per production cycle was unaltered. Thus at the farm level, the annual number of juveniles stocked and annual feed consumption decreased. The effect of $\sigma_{HW}$ on the average sales price is illustrated in Fig. 2: less variation led to more sales in size category 300 to 400 g (€4.52/kg) at the expense of sales in size category 200 to 300 g (€4.15/kg). Production results were unaltered by a change in $\sigma_{HW}$.

Economic values are in Table 7, which shows that the economic value of $\sigma_{HW}$ was similar for both levels of genetic change.

**Table 7:** Economic values of traits for gilthead seabream

Trait	Baseline trait level (trait unit)	Genetic change (trait unit)	Economic value [€ (kg production)^−1^ (trait unit)^−1^]
$TGC$ ^a^	12.6	+0.95	0.40
$TFC$ ^b^	8.25	−0.55	−0.45
$M$ ^c^	0.0300	−0.016	−7.7
$\sigma_{HW}$ ^d^	60	−4.2	−0.0011
$\sigma_{HW}$	60	−8.5	−0.0010

^a^Thermal growth coefficient [${\text{g}}^{2/3}$ /(day degrees · 1000)]

^b^Thermal feed intake coefficient [${\text{g}}^{0.544}$ /(day degrees · 1000)]

^c^Mortality rate (%/day)

^d^Standard deviation of harvest weight (g)

### Comparison of economic values from the bio-economic model and the profit equation

Table 8 shows that, for the simplified production system, the economic values derived from the bio-economic model and the profit equation were similar.

**Table 8:** Economic values derived from the bio-economic model and profit equation

Trait	Baseline trait level (trait unit)	Genetic change (trait unit)	Economic value [€ (kg production)^−1^ (trait unit)^−1^]	
			Bio-economic model	Profit equation
$BW_{{t_{s,h} }}$ ^a^	400	43.6	0.0074	0.0072
$CFI_{{t_{s,h} }}$ ^b^	713	−80.0	−0.0031	−0.0032
$S_{{t_{s,h} }}$ ^c^	85.1	7.66	0.016	0.019

^a^Harvest weight (g)

^b^Cumulative feed intake at harvest (g)

^c^Survival at harvest (%)

## Discussion

### Validity of the bio-economic model

For the simplified production system, the profit equation and the bio-economic model return similar economic values, which confirm the validity of the bio-economic model. To further validate the bio-economic model, production results were compared to those of other studies. FCR (Table 4) was within the range of 1.5 to 2 reported by Sola et al. [44] but considerably lower than the 2.3 value reported by EAS-EATiP [45]. Overall survival was 85%, which is within the range reported by EAS-EATiP [45]. A comparison with a cost-breakdown for large-scale production of gilthead seabream and European seabass (*Dicentrarchus labrax*) is in Table 9 [17]. In the FAO data, variable costs are higher, largely because labor, energy, and medicines and veterinary services were not considered to be variable costs in our study. Trends in the increase in productivity per person [46] support the assumption that labor should be treated more as a fixed than a variable cost. Medicine costs may vary, but veterinary costs are likely to be fixed per farm. Energy costs are to a larger extent determined by farm layout than by realized production and thus can be considered as fixed. Altogether, total variable costs may have been slightly underestimated in our study, but FCR and overall survival matched well to current industry standards.

**Table 9:** Cost-breakdown for gilthead seabream production

Item	Proportion of total costs (%)	
	Our study^a^	Barazi-Yeroulanos [17]
Feed	50	48
Juveniles	13	11
Marketing (incl. packing)	7	18
Labor	–	3
Energy	–	4
Medicines and veterinary services	–	2
Other	–	4
Total variable costs	70	89
Total fixed costs	–	11

^a^Relative to revenues

### Breeding goal

In the breeding goal, $TGC$, $TFC$, and $M$ are equivalent to respectively $BW_{{t_{s,h} }}$, $CFI_{{t_{s,h} }}$, and $S_{{t_{s,h} }}$, when $BW_{{t_{s,0} }}$ is much smaller than $BW_{{t_{s,h} }}$ (Appendixes 1, 2). When the sum of effective temperatures is the harvest criterion, one $\sigma_{A}$ change in $TGC$, $TFC$, and $M$ led to changes in $BW_{{t_{s,h} }}$, $CFI_{{t_{s,h} }}$, and $S_{{t_{s,h} }}$ that were very similar to the $\sigma_{A}$ of these traits (Table 8), which demonstrates their equivalence. If the economic values of $TGC$, $TFC$, and $M$ were calculated for the sum of effective temperatures instead of harvest weight as the harvest criterion, they would be slightly lower for $TGC$ [0.34€ (kg production)^−1^ (${\text{g}}^{0.544}$/(day degrees · 1000))^−1^] and unaltered for $TFC$ and $M$. In agreement with Wilton and Goddard [47], economic values were similar for both harvest criteria. Although both sets of traits are equivalent in the breeding goal, there are pros and cons to each one. $BW_{{t_{s,h} }}$ is commonly used as a selection criterion and thus its use in the breeding goal is straightforward. However, $BW_{{t_{s,h} }}$ is a management parameter that is strongly influenced by the growing period and temperature regime. $TGC$ corrects for heterogeneity in stocking weight, growing period, and temperature regime, and, therefore, allows for a better comparison of breeding values across conditions than $BW_{{t_{s,h} }}$ [11, 24, 25].

FCR could be used as an alternative to $TFC$ in the breeding goal. An advantage of feed intake compared to FCR is that it relates directly to feed costs [48]. An advantage of FCR is that it illustrates the effect of improvement in efficiency on, for example, environmental impacts, as in Besson et al. [49]. Feed intake is often considered more appropriate as a breeding goal trait than FCR [18, 19], with a common argument that traits expressed as ratio’s are disadvantageous in animal breeding [19]. Selection for a ratio, e.g. FCR, results in a lower selection response than selection for both components of the ratio, e.g. feed intake and growth [50]. However, in the same way that FCR is a ratio, growth is the ratio of feed intake to FCR, thus a breeding goal that includes both growth and FCR is equivalent to a breeding goal that includes growth and feed intake. The economic value of growth depends on which other trait, feed intake or FCR, is included in the breeding goal [48].

### Economic values

Bio-economic models and profit equations are both suitable to derive economic values. An advantage of a profit equation compared to a bio-economic model is its simplicity. However, its applicability is limited to specific situations, because environmental conditions are ignored. For example, the profit equation cannot be used to derive economic values for a range of temperature regimes, as was done in Besson et al. [11] by using the bio-economic model. Such properties may be of particular interest for breeding programs that aim at supplying many farms. In addition, alternative constraints on production output such as oxygen availability cannot be dealt with by the profit equation but can be incorporated in the bio-economic model, as discussed later. Furthermore, the profit equation is rigid in terms of trait definition, which has led to the false assumption that harvest weight changes following genetic improvement, whereas in the bio-economic model genetic improvement of growth rate leads to a reduction in the growing period.

From a profit function, economic values can be computed from either its partial derivative with respect to trait level, or from an increase or decrease in trait level relative to the current mean. In this study, simulated changes in trait levels correspond to desired directions of genetic change of one genetic standard deviation. However, for a non-linear profit function, Goddard [51] demonstrated that economic values that maximize profit in the next generation may depend on selection responses. Dekkers et al. [52] showed that economic gain is slightly higher when economic values are derived as the partial derivative of a non-linear profit equation at the genetic level of the next generation than at the genetic level of the current generation. This implies two things:

1. Economic values are closer to optimum when the simulated change in trait level resembles its expected rather than its desired direction.
2. Economic values are closer to optimum when the simulated change in trait level equals the difference between trait levels in the current and next generation than when it is the partial derivative at current genetic levels.

In our study, expected and desired directions of change in trait levels were identical, except for $TFC$, which may increase in practice due to its genetic correlation with $TGC$ [34]. A genetic standard deviation generally provides a better proxy for the difference between trait levels in the current and next generation than an infinitesimal change, and hence will result in an economic value that is closer to optimum than the conventional partial derivative at the current trait level.

This is the first time that economic values have been derived for uniformity in aquaculture, here expressed as $\sigma_{HW}$. In recent years, there has been increasing interest to improve uniformity [40, 53–55]. Improvement in uniformity affects average sales price and reduces the need for size-grading. However, for seabream production, reducing the need for size-grading would not result in major cost-savings, because seabream is size-graded only once during grow-out. Thus, a potential effect on grading frequency was excluded from the economic value of uniformity. Furthermore, uniformity has been suggested to affect feed intake, growth rate and mortality [56, 57]. Economic consequences of changes in other traits were accounted for in their respective economic values. By exploiting genetic correlations, selection for uniformity may be used to improve the other traits in the breeding goal.

### Application to other aquaculture species

In its current form, the bio-economic model can be easily applied for the derivation of economic values for other species produced in systems where stocking density limits production output, such as cages and flow-through tanks. This would require different values for the coefficients of Table 1, maximum stocking density, stocking and harvest weight, and input and output prices. Equations 4 and 5 require some species-specific modifications, such as alternative values for exponents 2/3 and 3/2 in Eq. 4 [58] or a different minimum temperature for growth.

Adaptations to the model are required for species that are reared in production systems for which constraints on production output are different, such as recirculating aquaculture systems and ponds. When the constraint on production output is different from stocking density, the number of fish stocked per production cycle (Eq. 9) is determined by other parameters. For recirculating aquaculture systems, treatment capacity of the biofilter can be a constraint on production output [12]. In this case, daily nitrogen excretion by fish is the parameter that determines the number of fish stocked per production cycle. Daily nitrogen excretion by fish can be predicted from the difference between daily feed consumption and daily gain in bodyweight, as described in Besson et al. [12]. In both cages and ponds, oxygen availability can be a constraint on production output. In this case, daily oxygen consumption per fish is the parameter that determines the number of fish stocked per production cycle. Daily oxygen consumption per fish can be predicted from daily feed consumption and daily gain in bodyweight, as described in Besson et al. [11]. With the above modifications, the same bio-economic model was applied for the derivation of economic values for African catfish produced in recirculating aquaculture systems [12], European seabass produced in cages [11], gilthead seabream produced in cages (this study), turbot produced in tanks (unpublished results), and Nile tilapia produced in ponds (unpublished results).

## Conclusions

We developed a bio-economic model to derive economic values for a wide range of aquaculture species. Its validity was confirmed by the comparison to a profit equation for a simplified production system and by comparison of the production results to those of other studies. Application of the bio-economic model to gilthead seabream resulted in economic values for $TGC$, $TFC$, $M$, and $\sigma_{HW}$. $TGC$ was the most important trait to improve, followed by $TFC$ and $M$. The effect of $\sigma_{HW}$ on gross margin was small.

## Appendices

### Appendix 1: Genetic variation in *TGC*

Genetic variation in $TGC$ depends on genetic variation both in $BW_{{t_{s,0} }}$ and $BW_{{t_{s,h} }}$:

25 $$Var\left( {A_{TGC} } \right) = Var\left( {1000 \cdot \frac{{A_{{BW_{{t_{s,h} }} }}^{2/3} - A_{{BW_{{t_{s,0} }} }}^{2/3} }}{{\mathop \sum \nolimits_{{i = t_{s,0} }}^{{(t_{s,h} ) - 1}} \left( {T_{i} - 12} \right)}}} \right),$$

where $A_{TGC}$ is the genotype for $TGC$, $A_{{BW_{{t_{s,h} }} }}$ is the genotype for harvest weight, and $A_{{BW_{{t_{s,0} }} }}$ is the genotype for stocking weight. Equation 25 can be rewritten as:

26 $$\begin{aligned} Var\left( {A_{TGC} } \right) & = \left( {\frac{1000}{{\mathop \sum \nolimits_{{i = t_{s,0} }}^{{(t_{s,h} ) - 1}} \left( {T_{i} - 12} \right)}}} \right)^{2} \cdot \left( {Var\left( {A_{{BW_{{t_{s,h} }} }}^{2/3} } \right) + Var\left( {A_{{BW_{{t_{s,0} }} }}^{2/3} } \right)} \right. \\ & \quad \left. { - 2 \cdot cov\left( {A_{{BW_{{t_{s,h} }} }}^{2/3} ,A_{{BW_{{t_{s,0} }} }}^{2/3} } \right)} \right). \\ \end{aligned}$$

Because $BW_{{t_{s,0} }}$ is much smaller than $BW_{{t_{s,h} }}$, $Var\left( {A_{{BW_{{t_{s,0} }} }}^{2/3} } \right) - 2 \cdot cov\left( {A_{{BW_{{t_{s,h} }} }}^{2/3} ,A_{{BW_{{t_{s,0} }} }}^{2/3} } \right)$ is much smaller than $Var\left( {A_{{BW_{{t_{s,h} }} }}^{2/3} } \right)$ [59]. Thus, Eq. 26 can be reduced to Eq. 15.

### Appendix 2: Genetic variation in cumulative feed intake at harvest

The regression coefficient of the genotype for $$CFI_{{t_{s,h} }}$$($$A_{{CFI_{{t_{s,h} }} }} )$$ on the difference between $$A_{{BW_{{t_{s,h} }} }}$$ and $$A_{{BW_{{t_{s,0} }} }}$$, can be calculated as:

27 $$b\left( {A_{{CFI_{{t_{s,h} }} }} ,A_{{BW_{{t_{s,h} }} }} - A_{{BW_{{t_{s,0} }} }} } \right) = r_{A} \cdot \sqrt {\frac{{Var\left( {A_{{CFI_{{t_{s,h} }} }} } \right)}}{{Var\left( {A_{{BW_{{t_{s,h} }} }} - A_{{BW_{{t_{s,0} }} }} } \right)}}} ,$$

where $b\left( {A_{{CFI_{{t_{s,h} }} }} ,A_{{BW_{{t_{s,h} }} }} - A_{{BW_{{t_{s,0} }} }} } \right)$ is the regression coefficient, and $r_{A}$ is the genetic correlation coefficient. For the regression of $A_{{CFI_{{t_{s,h} }} }}$ on $A_{{BW_{{t_{s,h} }} }} - A_{{BW_{{t_{s,0} }} }}$, the intercept corresponds to feed consumption to meet maintenance energy requirements ($CFI_{maintenance}$) at zero growth. Assuming a digestible energy content of the diet of 17 kJ/g, $CFI_{maintenance}$ is calculated as [26]:

28 $$CFI_{maintenance} = \mathop \sum \limits_{{i = t_{s,0} }}^{{t_{s,h} }} 47.89 \cdot (BW_{i} /1000)^{0.80} /17 = 20\;{\text{g}} .$$

For $A_{{BW_{{t_{s,h} }} }} - A_{{BW_{{t_{s,0} }} }} = 400 - 4.4 = 395.6\;{\text{g}}$, $A_{{CFI_{{t_{s,h} }} }}$ equals $1.80 \cdot 395.6 = 713\;{\text{g}}$, where 1.80 is the biological FCR (Table 4). The regression coefficient can thus be approximated as (713 − 20)/395.6 = 1.75 and Eq. 27 can be rewritten as:

29 $$1.75 = r_{A} \cdot \sqrt {\frac{{Var\left( {A_{{CFI_{{t_{s,h} }} }} } \right)}}{{Var\left( {A_{{BW_{{t_{s,h} }} }} } \right) + Var\left( {A_{{BW_{{t_{s,0} }} }} } \right) - 2 \cdot cov\left( {A_{{BW_{{t_{s,h} }} }} ,A_{{BW_{{t_{s,0} }} }} } \right)}}} .$$

Because $BW_{{t_{s,0} }}$ is much smaller than $BW_{{t_{s,h} }}$, $Var\left( {A_{{BW_{{t_{s,0} }} }} } \right) - 2 \cdot cov\left( {A_{{BW_{{t_{s,h} }} }} ,A_{{BW_{{t_{s,0} }} }} } \right)$ is much smaller than $Var\left( {A_{{BW_{{t_{s,h} }} }} } \right)$ [59]. Thus Eq. 29 can be reduced to Eq. 16.
